# Novel Therapeutic Approaches for Treating Moderate-to-Severe Atopic Dermatitis in Infants with Polyvalent Allergic Sensitisation

**DOI:** 10.34763/jmotherandchild.20263001.d-25-00037

**Published:** 2026-02-12

**Authors:** Maria Zofia Lisiecka

**Affiliations:** Department of Allergology, National Medical Institute of the Ministry of the Interior and Administration, 02-507, 137 Woloska Str., Warsaw, Poland

**Keywords:** skin barrier, Th2 immune modulation, pruritus reduction, microbial diversity, sensitisation, behavioural symptoms

## Abstract

The aim of this study was to evaluate the therapeutic efficacy of a comprehensive treatment approach in infants with moderate-to-severe eczema complicated by polyvalent allergic sensitisation. The methodology involved a randomised controlled trial that included 87 children aged 3 to 12 months. Participants were divided into two groups. The experimental group received combined therapy comprising pseudoceramides, a calcineurin inhibitor, and an oral synbiotic, while the control group received standard therapy with emollients and antihistamines. The results demonstrated that by the eighth week, patients in the experimental group exhibited a reduction in SCORAD index of more than 60% (to 21.4 ± 6.5); a 2.7-fold decrease in pruritus severity according to the VAS scale (to 2.9±1.3); and a 61% reduction in POEM score (to 7.3). This was accompanied by improved sleep, reduced irritability, and decreased behavioural disturbances. The control group showed less-pronounced positive dynamics. Immunological changes included a significant decrease in total and specific IgE levels, as well as a marked reduction in IL-4 and IL-13 concentrations, indicating attenuation of the Th2-mediated immune response. The prevalence of severe sensitisation decreased by 20%. Microbiota analysis demonstrated increased Bifidobacterium spp. abundance (+89%; p = 0.011); higher alpha-diversity (Shannon index + 27%; p = 0.004); and reduced E. coli levels (−36%; p = 0.018), suggesting restoration of microbial balance. Thus, this combined therapy demonstrated significant superiority, providing clinical improvement, suppression of the Th2 response, and normalisation of gut microbiota in infants with atopic dermatitis and multiple allergic sensitisation.

## Introduction

Atopic dermatitis (eczema) in infants remains one of the most prevalent and socially significant forms of chronic inflammatory skin disorders in early childhood. Since 2016, a steady increase in incidence has been observed, particularly in highly developed and urbanised regions. The disease is not only prevalent but also often severe, with symptoms such as intense pruritus, sleep disturbances, emotional lability, and chronic relapse. These manifestations significantly impair the quality of life of both affected infants and their families. A major aggravating factor is the presence of polyvalent allergic sensitisation, which complicates the condition’s clinical presentation and limits the efficacy of standard therapies. These developments underscore the need for novel therapeutic strategies that effectively alleviate both the symptoms and the immune/microbiological dysregulation associated with the condition.

A distinct challenge in the treatment of infantile eczema lies in the limited efficacy of conventional therapies in cases of multiple allergic sensitisation [1]. The use of emollients and antihistamines alone in such scenarios yields suboptimal outcomes, particularly in moderate-to-severe inflammatory presentations. K. Makowska et al. [2] found that monotherapy for atopic dermatitis with polyvalent sensitisation provides only transient symptom relief, with immunopathological processes remaining active, which predisposes patients to early relapse. The authors emphasise that a combined therapeutic approach is warranted in such cases, given the multifactorial nature of the disease’s pathogenesis.

Inadequate correction of skin barrier dysfunction represents another significant obstacle to effective treatment [3, 4]. Structural abnormalities in the epidermal lipid layer of infants with atopic dermatitis increase allergen penetration and perpetuate chronic inflammation. Research by E. Alska et al. [5] demonstrated that in such cases, standard therapies based on conventional emollients fail to sufficiently restore skin barrier function. The authors conclude that the use of components that structurally mimic natural ceramides holds promise for more comprehensive restoration of epidermal homeostasis.

The intensity of the Th2 immune response, and its role in the pathogenesis of eczema in infants with polyvalent allergic sensitisation, also requires close attention. A study by R. Fornal et al. [6] revealed elevated expression of IL-4 and IL-13 cytokines in this patient cohort, correlating with patients’ clinical severity. However, attempts to modulate this pathway using systemic immunosuppressants in young children have been limited by the high risk of adverse effects. The authors highlight the need for safer topical agents with immunomodulatory properties.

Disruptions in gut microbiota represent a key factor in the development and perpetuation of allergic conditions in children [7,8,9]. According to an analysis by J. Chalmers et al. [10], infants with eczema and polyvalent allergic sensitisation exhibit reduced alpha-diversity of microbiota, decreased abundance of beneficial *Bifidobacterium* spp., and an increase in opportunistic pathogens such as *Escherichia coli*. The authors underscore that restoring microbial balance may serve as a crucial adjunct therapy, particularly through the use of synbiotics, which combine probiotic strains and prebiotic substrates.

The unsatisfactory dynamics of total and specific IgE level reduction during standard treatment also presents a significant challenge. H.O. Skjerven et al. [11] demonstrated that infants with pronounced sensitisation maintain elevated IgE levels even after a course of baseline therapy; this is associated with a high risk of disease chronicity. The authors conclude that targeting the immune component of the disease is key to a comprehensive therapeutic approach.

Behavioural disturbances and sleep impairment in infants with severe eczema remain an underappreciated yet significant factor worsening patients’ prognosis. H. Kader et al. [12] found that persistent pruritus and inflammation lead to irritability, disrupted sleep patterns, and difficulties in daily child care. Despite this, most treatment regimens do not include interventions aimed at rapid symptom relief. The authors emphasise the importance of a multifaceted, comprehensive approach.

Another unresolved issue is the insufficient duration of clinical remission following treatment discontinuation. The study by S. Sun et al. [13] showed that children receiving only conventional therapy experienced symptom relapse as early as 3–4 weeks after treatment cessation. The authors highlight that effective therapy should not only alleviate acute manifestations, but also ensure sustained clinical and immunological improvement.

There is also a need for therapeutic strategies tailored to the specificities of infancy. M. Kelleher et al. [14] emphasise that pharmacokinetic and immunological parameters in infants under one year of age necessitate a specialised approach to treatment selection, as standard dosages and medications used in older children are not always effective or safe in infants. This underscores the importance of developing tailored treatment regimens that account for age-specific characteristics.

Given these knowledge gaps and the limitations of existing approaches, there is a pressing need for novel therapeutic strategies that provide multi-component modulation of key pathogenetic mechanisms of the disease. Unlike previous studies, which have focused on isolated interventions, this study evaluates a multicomponent therapeutic regimen combining topical skin barrier restoration, local immunosuppression, and oral synbiotic supplementation. The aim of this study was to evaluate the clinical and immunological efficacy of combination therapy in infants with eczema complicated by polyvalent allergic sensitisation. The study objectives included an analysis of skin manifestation dynamics, assessment of immune response parameters, and evaluation of microbiota status under conditions of comprehensive therapeutic intervention.

## Materials and methods

The study was conducted from January to September 2024 at the Department of Dermatology, Medical University of Warsaw, Poland, under both inpatient and outpatient settings. The study enrolled 87 infants (44 boys and 43 girls) aged 3 to 12 months with clinically confirmed diagnoses of moderate-to-severe atopic dermatitis based on the diagnostic criteria of J.M. Hanifin and G. Rajka [15] in the presence of concomitant allergic conditions, including food sensitisation, respiratory hyperreactivity, and a positive family history of atopy. Patients with congenital immunodeficiencies, systemic infections and acute dermatoses, as well as those who had received immunosuppressive therapy within 30 days prior to the study, were excluded. Written informed consent was obtained from all participants’ parents.

Participants were randomised into two groups. The experimental group (n = 45) received combination therapy consisting of topical application of pseudo-ceramide-based cream (Curel Intensive Moisture Cream, Japan) twice daily; a calcineurin inhibitor (Protopic 0.03%, Japan) once daily on affected areas; and a daily oral dose of 0.3 mL of a synbiotic (Alflorex Baby Drops, Ireland). The control group (n = 42) received standard therapy: emollients (Lipikar Baume AP + M, France) three times daily, and an antihistamine (Zyrtec, Switzerland) at an age-appropriate dose at bedtime. The treatment duration was eight weeks. Both groups also received individualised hygiene and dietary recommendations based on allergy testing results.

Clinical assessments were performed at weeks 0, 4, and 8, and the impact of atopic dermatitis on patients’ quality of life was measured using the following validated scales: SCORing Atopic Dermatitis (SCORAD) for quantitative evaluation of skin symptom severity; Visual Analogue Scale (VAS) for self-assessment of itch intensity and irritability levels; and Patient-Oriented Eczema Measure (POEM). Assessment of allergic sensitisation included skin-prick testing with a standard allergen panel (Stallergenes Greer, France); determination of total and allergen-specific IgE levels in blood serum using an enzyme-linked immunosorbent assay (ELISA) with the ImmunoCAP system (Sweden); and molecular allergy diagnostics employing the multiplex component-based ALEX^2^ Allergy Explorer panel (Austria). To analyse gut microbiota, 16S rRNA gene sequencing of faecal samples was performed using high-throughput sequencing on the Illumina MiSeq platform (USA). Bioinformatic processing of the obtained data was conducted in the QIIME 2 environment (USA).

Additionally, levels of inflammatory cytokines (TNF-α, IL-5, IL-13, IL-4) in blood plasma were assessed through multiplex analysis with the Luminex MAGPIX platform (USA). Blood sampling was performed after overnight fasting during the first and eighth study visits, and collected serum samples were stored at −80 °C until analysis. All procedures and interventions were carried out in accordance with the principles of the Declaration of Helsinki and the standards of Good Clinical Laboratory Practice (GCLP). The study protocol was reviewed and approved by the Bioethics Committee of the National Medical Institute of the Ministry of the Interior and Administration, Warsaw, Poland (Approval No. NMIMIA-DER-2023-12). All procedures were conducted in compliance with the Declaration of Helsinki. Written informed consent was obtained from the parents or legal guardians of all participating infants prior to their inclusion in the study.

Statistical analysis of the data was conducted using IBM SPSS Statistics v.28 (USA). The Shapiro-Wilk test was employed to assess the normality of data distribution. Comparisons between groups were made using Student’s t-test for independent samples and the Mann-Whitney U test for cases of non-normal distribution. Intragroup changes were analysed using the paired t-test. Differences in categorical data were assessed using the χ^2^ test or Fisher’s exact test as appropriate. Correlation analysis was performed using Spearman’s rank correlation coefficient. Differences were considered statistically significant at p < 0.05. The p-value was calculated using Student’s t-test for independent samples, as the distribution of SCORAD values at this stage conformed to normality according to the Shapiro-Wilk test.

## Results

During the eight-week observation period, marked differences in the dynamics of clinical parameters were observed between the experimental and control groups. At the baseline (week 0), the SCORAD, VAS, and POEM scores were comparable in both groups, confirming sample homogeneity and the absence of initial bias. By week 4, the experimental group showed showed significant improvement across all clinical parameters, with the most notable decrease observed in the SCORAD index. The positive trend continued through week 8, when both SCORAD and POEM scores approached the lower range of moderate disease severity, and VAS values indicated a marked reduction in pruritus intensity. The control group also demonstrated improvement, although changes were less pronounced. These differences were particularly evident upon analysis of standard deviations and interquartile ranges (Table 1).

**Table 1.** Mean values of SCORAD, VAS, and POEM scores at weeks 0, 4, and 8 in both groups.

**Parameter**	**Time (weeks)**	**Experimental group (n=45)**	**Control group (n=42)**
SCORAD	0	54.3 ± 6.1	53.9 ± 5.7
	4	35.6 ± 7.8	45.2 ± 6.4
	8	21.4 ± 6.5	38.1 ± 6.9
VAS (pruritus)	0	7.8 ± 1.1	7.7 ± 1.3
	4	4.3 ± 1.6	6.2 ± 1.4
	8	2.9 ± 1.3	5.1 ± 1.6
POEM	0	18.7 ± 3.9	18.5 ± 4.1
	4	11.4 ± 3.5	15.6 ± 3.7
	8	7.3 ± 2.6	13.1 ± 3.4

*Source: compiled by the author.*

A detailed analysis of the dynamics of the SCORAD, VAS, and POEM clinical scales revealed significant differences in the efficacy of the applied therapeutic approaches in infants with atopic dermatitis complicated by polyvalent allergic sensitisation. At baseline, none of the three scales demonstrated statistically significant differences between the experimental and control groups, indicating initial comparability in clinical status, severity of dermatological symptoms, and subjective discomfort.

By the fourth week of treatment in the experimental group receiving combined therapy – which included pseudoceramides, a calcineurin inhibitor, and an oral synbiotic – a marked reduction in the mean SCORAD index was observed, exceeding 34% compared to baseline. This decline was accompanied by a statistically significant difference from the control group, where the reduction was only 16% (p = 0.009), reflecting the moderate efficacy of the standard treatment regimen. By the eighth week, the disparity between the groups had further increased; mean SCORAD values in the experimental group fell within the range characteristic of mild atopic dermatitis, whereas the control group’s scores remained indicative of moderate disease activity. The intergroup difference remained highly statistically significant (p < 0.001), confirming the sustained and pronounced effect of the combined therapy.

Concurrently, significant changes were noted in the subjective assessment of pruritus using the VAS scale. While all participants initially reported itch severity scores of 7–8 points, corresponding to significant discomfort (7–10 points on the VAS scale), by the fourth week, the experimental group’s score showed a reduction to 4.3 ± 1.6, indicating moderate pruritus (4–6 points). By the eighth week, the mean score further decreased to 2.9 ± 1.3, reflecting a transition to mild symptom severity (1–3 points) or near-complete resolution. The control group also exhibited a reduction in symptom intensity, albeit less pronounced; final scores reached 5.1 ± 1.6, which is still indicative of moderate discomfort. The experimental group also demonstrated a narrower range of individual scores, suggesting greater uniformity in therapeutic response under the combined intervention.

The POEM scale, which assesses the impact of dermatitis on the child’s daily activities and overall condition, revealed a similar trend, further supporting the superiority of the experimental protocol, with a 61% reduction in mean scores (from 18.7 to 7.3) compared to only a 29% regression (from 18.5 to 13.1) in the control group (p < 0.01). This is particularly significant in the context of evaluating infants’ quality of life, given its dependence on the severity of cutaneous symptoms, pruritus, sleep disturbances, and irritability levels. Participants in the experimental group exhibited improved general well-being, fewer nocturnal awakenings, and reduced distress, as recorded in parental questionnaires.

At the initial observation stage, both groups had comparable levels of total IgE, specific IgE to food and inhalant allergens, as well as the concentrations of key pro-inflammatory cytokines (IL-4, IL-5, IL-13), which confirms the presence of pronounced allergic inflammation in all participants. The concentrations of IL-4 and IL-13, as typical mediators of the Th2 response, exceeded physiological age-adjusted values by more than twofold, while the level of IL-5 reflected eosinophilic activity. After eight weeks of treatment, the experimental group exhibited a statistically significant reduction in total IgE (p < 0.05), as well as specific IgE to major allergens, particularly β-lactoglobulin. Furthermore, a marked decrease in IL-4 and IL-13 levels was observed, suggesting a decline in Th2 response activity and an overall attenuation of allergic inflammation. In the control group, similar parameters either remained unchanged or showed a weak downward trend without statistical significance. The level of TNF-α remained stable in both groups, likely due to the absence of systemic inflammation and the predominantly localised nature of cutaneous involvement (Table 2).

**Table 2.** Changes in IgE and cytokine (IL-4, IL-5, IL-13, TNF-α) levels before and after therapy.

**Parameter**	**Experimental group (before)**	**Experimental group (after)**	**Control group (before)**	**Control group (after)**
Total IgE (IU/mL)	187.4 ± 34.1	142.7 ± 30.6	183.9 ± 36.2	169.8 ± 35.7
Specific IgE to β-lactoglobulin	12.3 ± 4.7	7.8 ± 3.5	11.9 ± 4.2	10.6 ± 4.4
IL-4 (pg/mL)	18.1 ± 3.2	11.7 ± 2.8	17.8 ± 3.5	15.9 ± 3.3
IL-5 (pg/mL)	10.5 ± 2.6	8.9 ± 2.7	10.3 ± 2.4	9.8 ± 2.5
IL-13 (pg/mL)	22.9 ± 4.5	14.6 ± 3.9	23.1 ± 4.2	20.4 ± 4.1
TNF-α (pg/mL)	7.2 ± 1.8	6.9 ± 1.7	7.1 ± 1.9	6.8 ± 1.8

*Source: compiled by the author.*

The results of the immunological analysis revealed significant distinctions between the two study groups in the patients’ modulation of systemic allergic inflammation. Prior to treatment, total IgE concentrations in most participants exceeded age-adjusted norms by more than threefold, confirming pronounced activation of the humoral immune response, which is characteristic of atopic dermatitis. In the experimental group, following a course of combination therapy, a significant reduction in total IgE was recorded (from 187.4 ± 34.1 to 142.7 ± 30.6 IU/mL), suggesting decreased B-cell activity and inhibition of Th2-mediated antibody production. This reduction was statistically significant (p = 0.032). In the control group, by comparison, a similar trend did not reach significance, indicating that standard therapy had a more limited effect on humoral hyperreactivity.

IgE specific to β-lactoglobulin – one of the most significant food allergens in infants – also showed a notable decrease in the experimental group (from 12.3 ± 4.7 to 7.8 ± 3.5 IU/mL). In contrast, the control group exhibited only a minimal reduction (to 10.6 ± 4.4), without statistically significant differences. This is likely due to the absence of immunomodulatory components in the treatment regimen.

The cytokine profile also underwent significant changes. In the experimental group, IL-4 levels decreased from 18.1 ± 3.2 to 11.7 ± 2.8 pg/mL, while IL-13 levels declined from 22.9 ± 4.5 to 14.6 ± 3.9 pg/mL. Both mediators play a pivotal role in Th2-cell differentiation and stimulate IgE synthesis. Their regression thus may be interpreted as a sign of suppressed allergic activation and improved skin barrier function. IL-5, responsible for eosinophilic activity, exhibited a less pronounced reduction (from 10.5 ± 2.6 to 8.9 ± 2.7 pg/mL), with the difference failing to reach statistical significance. This may be attributed to physiological peculiarities in early infancy, during which the eosinophilic response is still developing. In the control group, IL-4 and IL-13 levels also demonstrated a downward trend, but the magnitude of the reduction was half that observed in the experimental group, and it was not accompanied by a statistically significant intergroup difference.

TNF-α, a mediator of non-specific inflammation, did not exhibit substantial dynamics in either group, reflecting the absence of systemic inflammatory dysregulation and confirming the cutaneous localisation of the pathological process. This parameter may serve as an internal control for the stability of patients’ systemic status.

The findings demonstrate that combination therapy exerts a modulatory effect on both the spectrum of allergic sensitisation and the composition of gut microbiota in infants with atopic dermatitis. At baseline, the vast majority of participants in both groups exhibited polysensitisation to food and inhalant allergens, confirmed by skin prick tests and molecular allergy diagnostics. However, only the experimental group showed a 20% reduction in severe sensitisation frequency and a decrease in the mean ALEX^2^ class after eight weeks of therapy, accompanied by a decline in specific IgE levels. These changes were not observed in the control group, where the sensitisation profile remained stable. Gut microbiome analysis also revealed significant disparities: the experimental group exhibited a statistically significant increase in *Bifidobacterium* spp. abundance (p = 0.011) and improved alpha-diversity according to the Shannon index (p = 0.004), whereas these shifts in the control group were minimal. Statistical analysis was performed using the Mann-Whitney U-test due to the non-normal data distribution. A concurrent reduction in *E. coli* proportion (p = 0.018) suggests the normalisation of microbiological balance. Collectively, these data indicate that combination therapy promotes both immune and microbiological stabilisation, which may represent a critical component in the pathogenetic management of infantile atopy (Table 3).

**Table 3.** Changes in Allergy Profile and Gut Microbiome Before and After Therapy.

**Parameter**	**Experimental group (before)**	**Experimental group (after)**	**Control group (before)**	**Control group (after)**
Proportion sensitised to ≥3 allergens (%)	77.8	57.8	76.2	72.4
Mean ALEX^2^ sensitisation class	3.4	2.1	3.3	3.0
Shannon Index (alpha-diversity)	2.71 ± 0.36	3.43 ± 0.41	2.68 ± 0.33	2.74 ± 0.35
Relative abundance of *Bifidobacterium* spp. (%)	9.8 ± 3.2	18.5 ± 4.1	10.1 ± 3.0	11.4 ± 3.5
Relative abundance of *E. coli* (%)	17.4 ± 4.5	11.2 ± 3.6	16.9 ± 4.8	15.8 ± 4.4

*Source: compiled by the author.*

Analysis of the allergological examination results and gut microbiota composition revealed complex intergroup differences, reflecting the systemic effects of the administered therapy. Both cohorts exhibited a high degree of polyvalent sensitisation at baseline, with over 75% of infants in each group displaying positive allergic reactions to three or more allergens. The most frequently recorded reactions were to cow’s milk proteins (β-lactoglobulin); ovalbumin; house dust mites (*Dermatophagoides pteronyssinus*); and cat epithelium (*Felis domesticus*). This sensitisation pattern is typical in infancy with a burdened atopic history and reflects early development of a hyperreactive immune profile.

Following eight weeks of therapy, however, the experimental group demonstrated a significant reduction in sensitisation severity – the proportion of children sensitised to ≥ 3 allergens according to the ALEX^2^ scale decreased by nearly 20%, whereas the control group showed only a 4% reduction, which did not reach statistical significance. The decrease in specific IgE levels was accompanied by reduced reactivity in skin-prick tests, particularly for food allergens. This finding may indicate the initial phase of clinical tolerance development. Such immunomodulatory effects may be attributed to the multifaceted action of the therapy, in which pseudoceramides restore skin barrier function, reducing transepidermal allergen penetration; the calcineurin inhibitor suppresses local inflammation; and the synbiotic modulates systemic immune responses via the gut-immune system axis.

Concurrently, the experimental group exhibited significant shifts in gut microbiota composition, as assessed by 16S rRNA sequencing. A statistically significant increase in the relative abundance of *Bifidobacterium* spp., particularly *B. longum* – associated with normal immune maturation and anti-inflammatory potential – was observed. Additionally, restoration of microbial alpha-diversity was noted (Shannon index increase from 2.71 to 3.43), and this serves as a positive prognostic marker of microbiotal resilience. The reduction in *E. coli* abundance, whose excessive presence often correlates with systemic inflammation, further confirms the correction of intestinal biocenosis. No such changes were recorded in the control group, highlighting the limited impact of standard therapy on microbiome homeostasis.

The presence of parallel changes in sensitisation profiles and microbiome in the experimental group indicates a potential role for gut microbiota in the formation of immune responses in infants. The results show that combination therapy can help modulate both immune and microbial parameters, which may be useful in the treatment of atopic dermatitis complicated by multifactorial allergic sensitisation. However, given the relatively short duration of the study and the lack of long-term follow-up, these findings should be interpreted with caution. Nonetheless, the results of this study underscore the necessity of incorporating targeted microbiome interventions into therapeutic protocols for this age and clinical cohort.

Analysis of the dynamics in POEM and VAS scores, which reflect the subjective perception of disease severity and the impact of symptoms on the infants’ daily activities, revealed significant differences between the two therapeutic approaches. At baseline, both parameters were elevated in both groups: the mean POEM score exceeded 18, indicating a substantial disease burden on quality of life, and the VAS itch score, as rated by the parents or caregivers, ranged between 7–8, reflecting pronounced discomfort in the infants. By the fourth week of therapy, the experimental group exhibited a significant reduction in both POEM (to 11.4) and VAS (to 4.3), with further decreases by the eighth week (7.3 and 2.9, respectively). In the control group, despite a positive trend, improvements were less pronounced: POEM decreased to 13.1, and VAS to 5.1. Participants in the experimental group demonstrated reduced frequency of night awakenings, crying, and irritability, as confirmed by parental questionnaires. These findings suggest that combined therapy exerts a more pronounced effect on subjective parameters directly influencing the quality of life of both infants and their families (Table 4).

**Table 4.** Dynamics of POEM and VAS scores (parental assessment) during therapy.

**Parameter**	**Time (weeks)**	**Experimental group**	**Control group**
POEM (score)	0	18.7 ± 3.9	18.5 ± 4.1
	4	11.4 ± 3.5	15.6 ± 3.7
	8	7.3 ± 2.6	13.1 ± 3.4
VAS (pruritus)	0	7.8 ± 1.1	7.7 ± 1.3
	4	4.3 ± 1.6	6.2 ± 1.4
	8	2.9 ± 1.3	5.1 ± 1.6

*Source: compiled by the author.*

The data obtained from POEM and VAS scales allowed for the evaluation of the therapy’s impact not only on clinical manifestations, but also on parental perception of symptoms, which is particularly important when working with an infant cohort. Over the eight-week observation period, it was noted that combined therapy – comprising topical skin barrier restoration, local immunosuppression, and oral microbiome intervention – significantly reduced itch severity, normalised behavioural manifestations, and improved children’s night-time sleep. During treatment, the mean POEM score decreased by 61% (from 18.7 to 7.3), corresponding to a shift from severe to mild or moderate impact of symptoms on daily life. In contrast, the control group showed only a 29% reduction (from 18.5 to 13.1), with values at week 8 remaining above thresholds indicative of clinically significant dermatitis-related quality-of-life impairment.

The VAS scale demonstrated a similar trend: in the experimental group, the severity of pruritus decreased by a factor of 2.7 (from 7.8 to 2.9 points), reflecting not only an objective reduction in symptoms, but also an improvement in infant comfort, including decreases in crying, irritability, sleep disturbances, and episodes of nocturnal awakening. In the control group, the reduction was more moderate (from 7.7 to 5.1 points), and subjective complaints persisted in the majority of parents. These differences were statistically significant (p < 0.01) and the effect showed greater sustainability in the experimental group, which is particularly important from the perspective of real-world clinical practice focused on the quality of life of patients and their families.

It is worth emphasising separately that in the questionnaires accompanying the POEM scale, parents of participants in the experimental group more frequently reported improvements in their child’s perceived emotional state, as well as decreases in the number of exacerbations and the need for additional care and symptomatic medications. This confirms that combination therapy influences not only biological parameters, but also daily functioning, creating the prerequisites for a more stable disease course in the future. Improvement in parental quality of life, mediated by the reduction in symptom severity in the child, also represents a significant clinical outcome in paediatrics. Thus, the combined approach demonstrated superiority in key subjective parameters, confirming its role as an effective strategy in the treatment of eczema within the context of multifactorial allergic pathology in early childhood.

## Discussion

The findings from clinical observation demonstrate the clear advantage of combination therapy over the standard approach in treating infants with atopic dermatitis complicated by polyvalent allergic sensitisation. The significant reduction in SCORAD, POEM, and VAS scores observed in the experimental group by week 8 indicates high efficacy for the therapeutic combination. Similar conclusions have been reported in studies by D. Palmer et al. [16] and E.L. Plummer et al. [17], who also documented clinically meaningful improvements in children’s condition when using a comprehensive approach combining topical intervention and systemic immunomodulation. This supports the hypothesis that cutaneous and behavioural indices are highly sensitive to multicomponent therapy.

The present study revealed that using pseudoceramides and a calcineurin inhibitor in combination with a synbiotic exerts a synergistic effect, ensuring both restoration of the skin barrier function and normalisation of the immune response. Studies by J. Armstrong et al. [18] and Z. Su and Y. Kang [19] primarily emphasise the local effects of such protocols, without finding significant systemic impact. However, the absence of data on cytokine profiles and microbiomic markers in their studies limits the validity of these conclusions. Conversely, the results presented in this study are supported by intergroup differences not only in clinical assessment scales, but also in immunological and microbiome-related criteria.

A significant decrease in IL-4 and IL-13 concentrations was observed in the experimental group participants, indicating modulation of the Th2-mediated immune response. These findings align with the observations of L. Bradshaw et al. [20] and T. Ito and Y. Nakamura [21], who described similar cytokine dynamics following the use of synbiotic complexes in early life. The reduction in IL-4 and IL-13 was correlated with decreased total IgE levels and specific sensitisation. Thus, the results of this study support the rationale for incorporating microbial components into therapeutic protocols for atopy. Analysis of the POEM scale revealed that combination therapy leads to a reduction in the symptomatic burden of the disease on daily activities, including behavioural and emotional aspects. Similar observations were reported by W. Wu et al. [22], as well as K. Yamamoto-Hanada and Y. Ohya [23], who noted that improvements in infant quality of life should be considered a key parameter of treatment efficacy. In the context of the present study, this is supported by a marked reduction in nocturnal discomfort and irritability in children from the experimental group.

However, it should be noted that several researchers, including E.L. Simpson et al. [24] and J.I. Silverberg et al. [25], have expressed scepticism regarding the necessity of adding probiotic or adjunctive components to standard therapies for atopic dermatitis. Their large-scale phase 3 clinical trials demonstrated that significant clinical improvements can be achieved using targeted topical agents, such as roflumilast cream 0.15% and tapinarof cream 1%, without the inclusion of microbial or systemic immunomodulatory supplements. Both studies reported robust reductions in eczema severity and pruritus indices (EASI 75 and VIGA-AD scores) with favourable safety and tolerability profiles. However, these investigations primarily focused on monotherapy with advanced anti-inflammatory topical formulations, and did not employ a multicomponent approach addressing both barrier and microbiome correction. In contrast, the present study utilised a combined protocol, achieving a synergistic effect through the complementary action of its components. The positive dynamics in VAS scores within the experimental group confirm a subjective reduction in pruritus, which is of particular significance in paediatric practice. According to Z. Wang et al. [26] and B. Liu et al. [27], pruritus is the primary factor contributing to behavioural disturbances and sleep disruption in infants with atopic dermatitis. In this study, a 63% reduction in VAS scores was accompanied by a decrease in nocturnal awakenings and crying episodes, indicating systemic improvement in condition.

Immunological parameters, particularly the reduction in β-lactoglobulin-specific IgE, demonstrate a pronounced immunomodulatory effect of the combination therapy, mediated through regulation of B-cell activity and suppression of allergen-specific antibody production. Given that β-lactoglobulin is one of the most clinically significant food allergens in infancy, a decrease in corresponding IgE levels may be regarded as an early marker of tolerance development. Studies by J. Anderson and T. Sivesind [28] and K. Hon et al. [29] emphasise the importance of allergen-specific responses as a key indicator of the initial stage of immune adaptation, associated with a reduced risk of progression from transient sensitisation to persistent allergic pathology. In the present study, the reduction in specific IgE was accompanied by a decrease in clinical symptoms, suggesting a correlation between immunological and clinical improvements. These results are consistent with the conclusions of the aforementioned authors, and they highlight the potential of combined intervention as a strategy capable of not only alleviating symptoms, but also reshaping immune architecture.

A sceptical stance regarding the efficacy of multicomponent therapy is taken by Y. Liu et al. [30] and X. Chen et al. [31], who emphasise that most effects of such interventions are transient and do not lead to sustained changes in the patient’s immune profile. However, the absence of microbiome analysis and cytokine profiling in their studies limits the interpretation of their findings. In contrast, the present study demonstrates a concurrent reduction in IgE and cytokines IL-4 and IL-13, as well as normalisation of microbiota, indicating a comprehensive and potentially long-term effect. In the experimental group, a significant increase in *Bifidobacterium* spp. abundance, and a rise in the alpha-diversity index, suggest the restoration of microbiome balance. Importantly, these results are consistent with the mechanisms described by M. Wang et al. [32] and S. Traidl et al. [33], who showed that modulation of the immune-microbiome axis, including reduction of IL-4 / IL-13 activity and restoration of beneficial bacterial populations, leads to alleviation of itching and improvement of skin barrier integrity in infants with atopic dermatitis. The observed increase in the number of *Bifidobacterium* spp. and the higher alpha diversity index in our experimental group confirm their findings, linking microbiome restoration with a reduction in allergic symptoms and improved skin homeostasis. Thus, our results not only agree with previous mechanistic data but also extend them, providing integrated clinical, immunological, and microbiological confirmation of the pathogenic role of dysbiosis in atopic dermatitis. These data confirm the targeted correction of the microbiome as a pathogenetically justified component of complex therapy.

On the other hand, the potential of *Patchouli* essential oil as a different therapeutic method for treating eczema symptoms in newborns and young children was examined by F. Deliana et al. [34]. According to their review, patchouli essential oil has anti-inflammatory, antibacterial, and antioxidant qualities that help lessen erythema, itching, and the severity of eczema overall. The scientists did, however, highlight the methodological shortcomings of the existing research, such as the lack of standardised dosages or long-term follow-up data, as well as inconsistencies in treatment methods. This demonstrates the potential of phytotherapeutic substances, but also the necessity of thorough clinical validation and safety assessment prior to the recommendation of such methods for general application in paediatric treatment.

P. Patra et al. [35] discusses the difficulties in identifying and treating atopic dermatitis and paediatric psoriasis, highlighting the intricate interactions between immune pathways, particularly the Th2/IL-4/IL-13 axis in atopic dermatitis and the Th17/IL-17/IL-23 axis in psoriasis. The scientists pointed out that if systemic immune dysregulation and epidermal barrier dysfunction are not treated simultaneously, clinical gains may be limited, even in cases when microbiome manipulation results in detectable physiological changes. Their results lend credence to the idea that integrated approaches that target both immune and barrier processes are necessary for effective therapy in children with chronic inflammatory dermatoses. However, in the present investigation, a significant decrease in sensitisation levels coincided with the normalisation of the microbiological profile, as evidenced by a fall in the mean ALEX^2^ class and a decrease in the percentage of children with polyvalent allergies. A direct and functionally important relationship between the microbiome and immune markers is suggested by the notable improvement in patients’ clinical status, including decreased pruritus, better sleep, and improved general behaviour. Therefore, the findings obtained demonstrate the effectiveness of an integrative approach that combines microbiome repair and immunomodulation, especially in cases of severe sensitisation where traditional therapy is insufficient.

The observed reduction in the proportion of children sensitised to three or more allergens in the experimental group confirms the impact of combined therapy on the degree of allergic response and demonstrates the potential for establishing a more tolerant immune profile. This decrease in sensitisation was also accompanied by a reduction in the mean ALEX^2^ class, indicating not only quantitative but also qualitative changes in the allergen sensitivity spectrum. These findings may suggest the initial stage of clinical tolerance development, particularly concerning food allergens, which are traditionally considered the most resistant to immunotherapy in infancy. They align with data from L. Pittet et al. [36] and R. Jaiswal et al. [37], who demonstrated a reduction in polyvalent sensitisation following microbiota correction and skin barrier improvement, underscoring the importance of an integrative impact on the skin-immune axis.

However, methodological and interpretive issues must be addressed. The trial was randomised; however, topical preparations, as well as the synbiotic’s administration route, made blinding participants and their caregivers impossible. In subjective outcome measures like VAS and POEM, which relied on parental reporting, this may have added performance or observer bias. Parental compliance with topical or oral medication may have affected adherence and efficacy between groups. To reduce bias, future studies should use blinded outcome assessment or digital therapy adherence monitoring. Environmental and behavioural factors like nutrition, humidity, and parental skin care may also explain group variations. Both groups got the same hygiene and food advice, but its execution may have affected clinical results. Even if randomisation balanced these effects, residual confounding remains. A brief eight-week follow-up period is another drawback. Gains in immunological and microbial normalisation were only measured in the short term, making their permanence questionable. Long-term investigations are needed to evaluate whether the decreases in IgE, IL-4, and IL-13 indicate temporary regulation or immunological reprogramming.

Though the sample size was large enough to identify substantial intergroup differences, it may still restrict generalisability. The geographically and ethnically homogeneous study participants may not adequately represent global genetic and environmental causes of atopic dermatitis. The absence of placebo control and double-blinding may increase expectation effects, especially for pruritus and irritation. Future research with a placebo group, or with similar treatment packaging and formulas varied only in active components, might improve internal validity. Despite these limitations, immunological and microbiological results support the clinical improvements, showing bias or behavioural confounding were not the main cause. Cytokine and IgE reduction and microbiome diversity restoration indicate a synergistic therapeutic mechanism.

In summary, the data from this study support the conclusion that the combined therapeutic approach is highly effective in treating atopic dermatitis in infants with pronounced allergic sensitisation. The obtained results demonstrate clinical, immunological, and microbiome improvements, indicating the systemic impact of the proposed protocol. The reduction in IgE levels, normalisation of the cytokine profile, decreased sensitisation, and restoration of microbiota confirm that further investigation and implementation of such therapy will be necessary in clinical practice. Promising future directions include assessing the long-term sustainability of the achieved effect, optimising the synbiotic composition, and expanding the inclusion age range.

## Conclusions

The present study demonstrated the clinical and immunological efficacy of combined therapy in infants with atopic dermatitis complicated by polyvalent allergic sensitisation. By the fourth week, the experimental group showed marked improvement in cutaneous symptoms, pruritus, and quality of life, and by the eighth week, reductions in SCORAD, VAS, and POEM exceeded 60%, compared to the merely moderate progress in the control group.

Combined therapy significantly reduced total and specific IgE, including IgE to β-lactoglobulin, and decreased concentrations of Th2-related cytokines (IL-4 and IL-13), indicating suppression of allergic inflammation and partial immune regulation. TNF-α levels remained stable, supporting the localised nature of the inflammatory process. The proportion of children with severe polyvalent sensitisation declined by 20%, and the mean ALEX^2^ class decreased notably, reflecting a shift toward a less-reactive allergic profile.

Microbiome assessment revealed favourable changes consistent with restoration of microbial balance, including greater *Bifidobacterium* spp. abundance and improved diversity in the microbiome. This supports the idea of gut-immune interactions being involved in disease modulation.

Overall, these findings support combined therapy as a pathogenetically justified and effective approach to managing infantile atopic dermatitis with complex allergic sensitisation. Further studies with extended follow-up are warranted to evaluate the durability of these clinical and immunological improvements and their potential role in promoting allergen tolerance.

## List of Abbreviations

ELISA: Enzyme-Linked Immunosorbent Assay
GCLP: Good Clinical Laboratory Practice
POEM: Patient-Oriented Eczema Measure
SCORAD: SCORing Atopic Dermatitis
VAS: Visual Analogue Scale
